# PAT: predictor for structured units and its application for the optimization of target molecules for the generation of synthetic antibodies

**DOI:** 10.1186/s12859-016-1001-1

**Published:** 2016-04-01

**Authors:** Jouhyun Jeon, Roland Arnold, Fateh Singh, Joan Teyra, Tatjana Braun, Philip M. Kim

**Affiliations:** Terrence Donnelly Centre for Cellular and Biomolecular Research, University of Toronto, Toronto, M5S 3E1 ON Canada; Department of Molecular Genetics, University of Toronto, Toronto, M5S 3E1 ON Canada; Department of Computer Science, University of Toronto, Toronto, M5S 3E1 ON Canada

**Keywords:** Structural domains, Protein domain, Protein sequence, Antibody target molecules, Synthetic antibody, Putative structural unit, Phage display

## Abstract

**Background:**

The identification of structured units in a protein sequence is an important first step for most biochemical studies. Importantly for this study, the identification of stable structured region is a crucial first step to generate novel synthetic antibodies. While many approaches to find domains or predict structured regions exist, important limitations remain, such as the optimization of domain boundaries and the lack of identification of non-domain structured units. Moreover, no integrated tool exists to find and optimize structural domains within protein sequences.

**Results:**

Here, we describe a new tool, PAT (http://www.kimlab.org/software/pat) that can efficiently identify both domains (with optimized boundaries) and non-domain putative structured units. PAT automatically analyzes various structural properties, evaluates the folding stability, and reports possible structural domains in a given protein sequence. For reliability evaluation of PAT, we applied PAT to identify antibody target molecules based on the notion that soluble and well-defined protein secondary and tertiary structures are appropriate target molecules for synthetic antibodies.

**Conclusion:**

PAT is an efficient and sensitive tool to identify structured units. A performance analysis shows that PAT can characterize structurally well-defined regions in a given sequence and outperforms other efforts to define reliable boundaries of domains. Specially, PAT successfully identifies experimentally confirmed target molecules for antibody generation. PAT also offers the pre-calculated results of 20,210 human proteins to accelerate common queries. PAT can therefore help to investigate large-scale structured domains and improve the success rate for synthetic antibody generation.

**Electronic supplementary material:**

The online version of this article (doi:10.1186/s12859-016-1001-1) contains supplementary material, which is available to authorized users.

## Background

Protein domains are fundamental units to study protein structure, conformation, function and evolution. A protein domain is generally defined as a structural unit which can fold independently and have their unique biological function [1], while their identification usually relies on their property of being conserved in evolution [2]. The identification of structural domains has become more prominent to engineer protein properties by experimental means [3], model protein structures using computational approaches [4] and determine 3D structures using X-ray crystallography and Nuclear Magnetic Resonance (NMR) [5]. Especially, identification of stable structural domain is a crucial first step to generate novel synthetic antibodies [6]. For these reasons, many approaches have been suggested to identify structural domains. In earlier work, Huang et al. implemented a method (DisMeta) to identify structured regions by excluding disordered regions [7], thereby implicitly (but not explicitly) detecting stably folded structures. Also, a number of methods have been developed to identify protein structural domains: Marsden et al. developed DomPred that predicts structural domains using the alignment of predicted secondary structures of a given target against secondary structures of known domains [8]. A number of *ab-initio* methods have also been attempted to structural domains. They incorporated position specific physico-chemical properties of amino acids, amino acid composition, relative solvent accessibility, as well as evolutionary information in the form of sequence profiles [9, 10]. While such approaches exist, there still is no efficient and integrative computational pipeline to identify structural domain for optimizing their likelihood of expression and folding. Furthermore, a user-friendly webserver to predict these targets is not available.

To address this need, we developed an integrated computational framework, PAT (Predictor for structural domains to design Antibody Target molecules), that can predict optimal structural domains. PAT automatically analyzes various structural properties, evaluates the folding stability, and identifies possible structured units in a given protein sequence. PAT identifies two types of structured regions with reliable boundaries. The first are traditional domains, i.e. strongly conserved stretches of protein sequence that usually adopt compact folds that are annotated in usual databases such as Pfam [2]. The others are putative structural units, i.e., parts of the protein that adopt stable folds but are not contained in current domain databases, presumably due to a lack of sequence conservation (unassigned regions). For the identification of putative structural units, PAT employs a novel scoring system by measuring the relevance of structural properties, integrating structural properties systematically, and generating target score that can represent folding stability of target molecules. PAT also provides users with the results of each intermediate calculation, including residue-specific evolutionary rate, disorderness, secondary structure, presence of trans-membrane and signal peptide, hydrophobicity, antigenicity, and compilation of primary amino acid sequences homologous to the query that can help further analyses of the user’s proteins of interest.

In this study, to show the wide application of structural domain prediction, we applied PAT to identify target molecules of synthetic antibodies. Synthetic antibodies are invaluable tools for the recognition of specific protein targets and have numerous applications in clinical studies and biological science [11]. Also, antibodies are applied to high-throughput proteome-wide studies to explore expression levels, subcellular localizations, and physical associations of target proteins [12]. It has been shown that proteins fragments that fold into stable structures are preferred as target molecules and consistently lead to high-affinity antibodies [6, 13]. Furthermore, these structural domains have been used as targets to produce affinity reagents and suitable constructs for antigen cell-surface display [14]. One of the major bottlenecks of synthetic antibody generation is the optimal identification and production of suitable antibody targets (sometimes referred to as antigens) since potential target proteins often fail to express or do not lead to high affinity binders [15]. In our proof-of-principle experiment, we showed that integrating structural properties of RNA-binding proteins (RBPs) can characterize protein regions that act as targets of synthetic antibodies [16]. In this study, we proved that PAT can be broadly applied to all protein families and effectively identify structural domains that can be target molecules for synthetic antibody generation.

## Implementation

### PAT overview

**Fig. 1:**
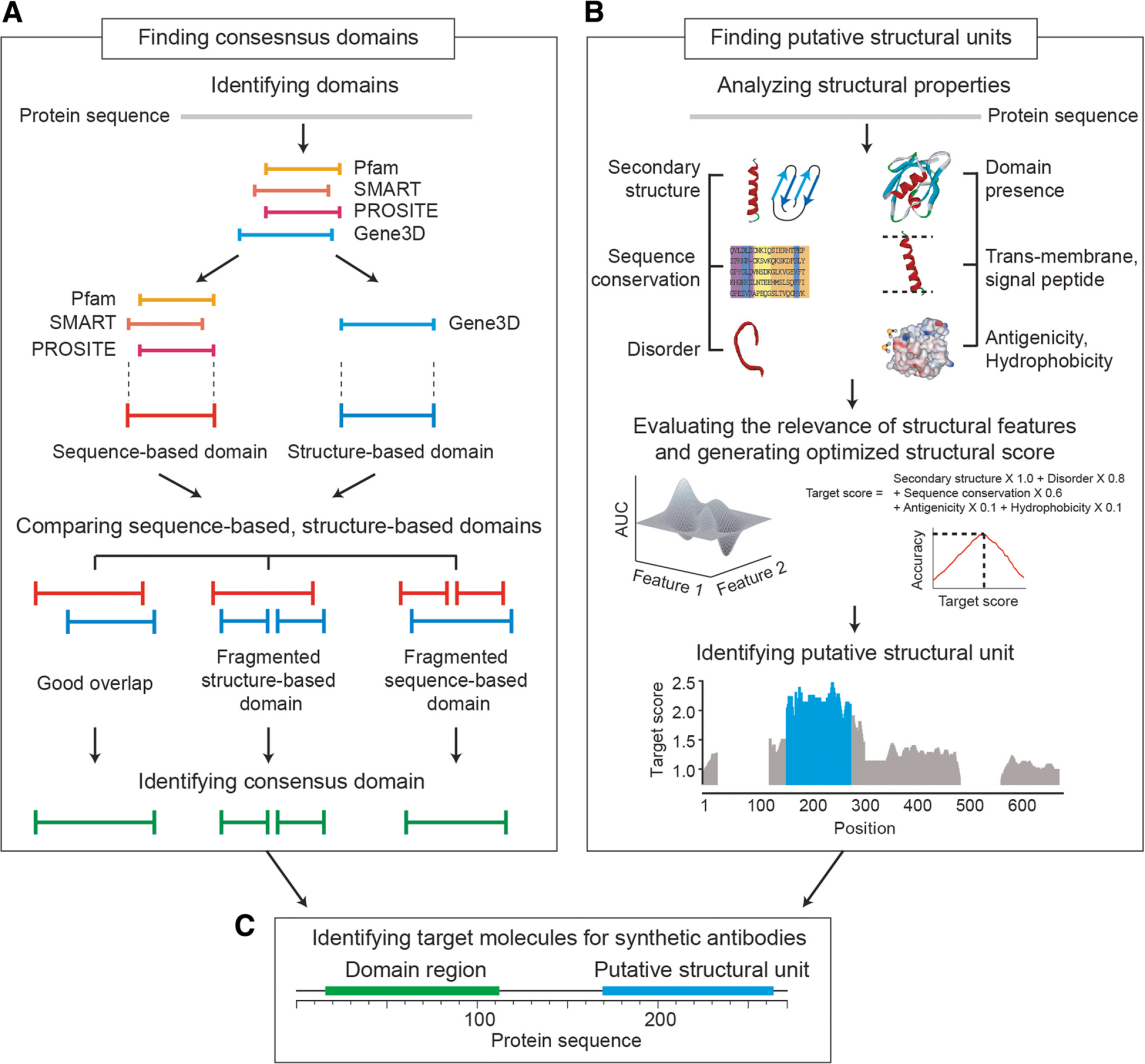
A flow scheme of PAT pipeline. **a** Procedure to find protein domain regions. **b** Procedure to find putative structural units. **c** Identification of two types of structured units

PAT is composed of two pipelines (Fig. 1). One pipeline characterizes protein domains, which are structurally compact and independent folding units, and optimizes their boundaries. The other evaluates the folding stability of putative structural units that have stable folds but are not covered in current domain databases.

### Identifying protein structured units

PAT integrates four domain databases to identify protein domains (Fig. 1a). First, PAT defines two types of domains (see Additional file 1 for details): sequence-based domains (from Pfam [2], SMART [17], and PROSITE [18]) and structure-based domains (from Gene3D [19]). Then, the sequence-based and structure-based domains are compared to find a consensus domain. We encounter three different cases: First, if one sequence-based domain maps to more than 50 % of one structure-based domain (we refer to this case as “good overlap”), the region that covers both types of domains is determined as a consensus domain. If several structure-based domains map to one sequence-based domain (“fragmented structure-based domain”) or vice versa (“fragmented sequence-based domain”), the structure-based domain is considered as a consensus domain. If a given protein only contains sequence-based domain annotations, the sequence-based domain is selected.

### Identifying putative structural units

Even in the absence of domain annotations, protein regions that show secondary structure, low levels of disorder, and sequence conservation in close species tend to adopt well-defined tertiary structures and can be appropriate target molecules for many areas of protein structure research as well as synthetic antibody generation [20–22]. Also, it has been shown that there is a positive correlation between soluble protein expressions yield and the number of contiguous hydrophobic residues and low complexity regions [23]. Furthermore, such protein regions tend to have novel structural folds with uncharacterized function implying that generating antibodies against structural putative units can be the first step to elucidate biological roles of novel structural folds. We thus developed a novel prediction algorithm that combines those sequence/structure-related features and devised a target score that can help to evaluate folding stability and expression of putative structural units (Fig. 1b, see Additional file 1 for details). For a given protein, PAT compiles information on secondary structure, known domain, trans-membrane region, signal peptide, residue-specific evolutionary rate, antigenicity, disorderness, and hydrophobicity. In order to integrate the information of individual features into a target score, we evaluated the relevance of each feature (weight) based on a grid-search over possible parameter space and optimized a scoring scheme. We did an exhaustive search of each feature weight and choose the set with optimum performance on the training set. As a training set, we used 164 proteins that have determined structures in the PDB database [24], but do not have any domain annotations in Pfam (Additional files 1 and 2). We considered these structural regions (16,430 residues) as a positive set and the rest of structural regions that do not have any structural information in 164 proteins (63,122 residues) as a negative set. The area under the Receiver Operating Characteristics (ROC) curve has been used to assess the performance of each combination of feature weights. The weights that yielded the best ROC (residue-wise) are selected as the best weight combination. The optimized target score is;

$$ \begin{array}{l}\mathrm{Targetscore} = \mathrm{Disorderness}\times 0.8 + \mathrm{Secondary}\ \mathrm{structure}\times 1.0 + \mathrm{Sequence}\ \mathrm{conservation}\times 0.6 + \\ {}\mathrm{Antigenicity}\times 0.1 + \mathrm{Hydrophobicity}\times 0.1\end{array} $$

This optimized score shows an area under the ROC curve of 0.68. This score reflects performance on the amino acid level (i.e., it is reflective of substantially higher accuracies at the protein level, when allowing for some boundary error). Next, PAT determines putative structural units that are enriched with high scoring residues. To do this, PAT employs a density grid clustering algorithm [25]. First, PAT divides the area of the protein into a number of “grids” of 5 residues and calculates an average target score of each grid. Then, the grid that has the highest average target score is defined as the center of the putative structural unit. Finally, the putative structural unit is extended as long as its target score is larger than a defined cut-off. At the target score cut-off of 0.52, PAT shows the best balanced accuracy (68.91 %), specificity of 62.94 and 74.88 % of sensitivity (Additional file 1: Figure S1). We use all putative structural units of a minimum length of more than 40 residues. As a result, PAT reports a set of structural domains including well-defined structural domains and putative structural units with their boundaries (Fig. 1c).

## Results and discussion

### Performance evaluation of PAT

We evaluated PAT predictions using three approaches. First, to ensure PAT provides reliable structural domain boundaries that can lead to successful protein expression and purification [26], we experimentally tested the efficiency of expression and purification of 210 predicted structural domains from PAT (the first pipeline in Fig. 1 and Additional file 3). These 210 domains represent 48 structural families that show wide range of domain size (55a.a ~ 438a.a) and sequence identity (average sequence identity is 8.95 %) distributions (Additional file 1: Figure S2). The experimental results show that 173 (82.38 %) were correctly expressed and 145 (69.05 %) were successfully purified (Table 1). Meanwhile, from the large-scale protein structure initiative, TargetTrack (version of Dec 07, 2012; [27]) reported that among 49,227 cloned targets, 32,904 (66.84 %) were expressed and 15,617 (31.72 %) were purified indicating that PAT outperforms current large-scale expression efforts.

**Table 1:** Comparative performance of PAT and TargetTrack

Progress	PAT^a^	TargetTrack
Number of targets	210	49,227
Expression	173 (82.38 %)	32,904 (66.84 %)
Purification	145 (69.05 %)	15,617 (31.72 %)

^a^Protein domains were expressed in 1.4 ml 2YT media at 30 °C overnight and the soluble his-tagged proteins were purified by affinity

**Fig. 2:**
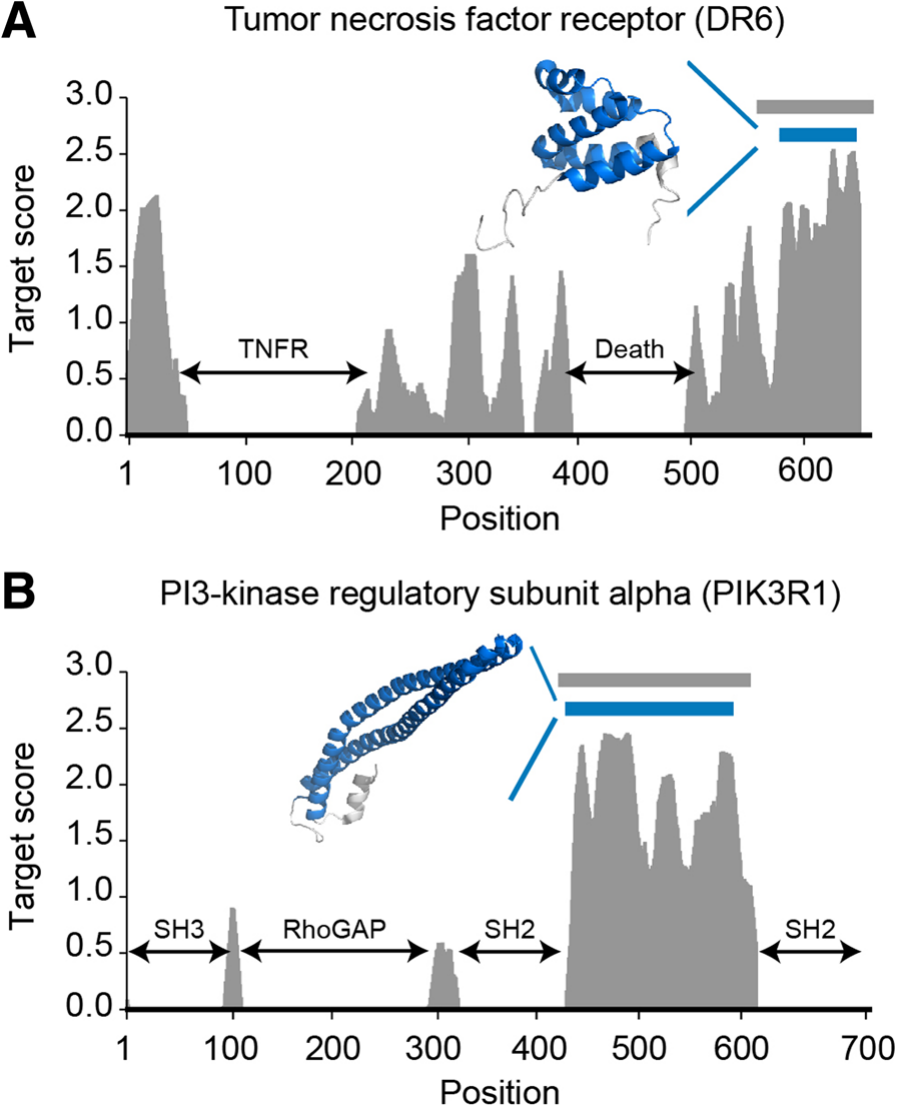
Identification of putative structural units. Putative structural units of (**a**) tumor necrosis factor receptor (DR6, PDB ID: 2DBH) and (**b**) phosphatidylinositol 3-kinase regulatory subunit alpha (PIK3R1, PDB ID: 2V1Y) are shown as blue bars. The structures (colored as blue on structures) represent putative structural units that correspond to blue bars in the graph. Gray bars represent the regions whose known structures are not listed as domains. Black arrows indicate protein domains. Since we excluded protein domain region when we calculate target scores, protein domain regions have target score of zero

Second, we examined the performance to identify putative structural units using an independent set of 20 protein structures that are not used for the optimization of PAT pipeline (Additional file 2). The 20 proteins include 3169 positions that are involved in putative structural regions (positive set) and 5489 positions that do not have domain annotations and known structures (negative set). As shown in Table 2, PAT correctly assigned 2370 of 3169 positive positions (74.79 % sensitivity) and 3601 of 5489 negative positions (65.60 % specificity). The PAT has an accuracy of 68.97 % and a precision of 55.66 %. Indeed, we found that PAT can capture well-defined structures. For example, in death receptor 6 (DR6), the protein region ranging from F576 to D645 was predicted as a putative structural unit. When we mapped this predicted region onto the known protein structure (PDB ID: 2DBH), the region covered a folded structure that is composed of 5 helices (Fig. 2a). In phosphatidylinositol 3-kinase regulatory subunit alpha (PIK3R1), PAT predicted the region ranging from V436 to R590 as a putative structural unit. This region is composed of two long helices forming a coiled-coil (Fig. 2b; PDB ID: 2V1Y). Furthermore, we computed the target scores of unexpressed constructs in TargetTrack (16,323 targets) as these would correspond to a proteins that are difficult to express and thus likely not stably folded. We then compared these scores with the scores of 184 known putative structural units (164 targets are used for training and 20 targets are used for testing of PAT pipeline). As shown in Additional file 1: Figure S3, the average target score of our set is about two times higher than the score of unexpressed targets with high statistical significance (*P*-value = 4.83 × 10^−25^). It suggests that PAT can identify appropriate structural units that would be well expressed and purified.

**Table 2:** Performance of PAT to identify putative structural units

	Success	
	Positive	Negative
Target score prediction		
Positive	2,370 (TP)	1,888 (FP)
Negative	799 (FN)	3,601 (TN)
Metrics		
Sensitivity (%)	74.79	
Specificity (%)	65.60	
Accuracy (%)	68.97	
Balanced accuracy (%)	70.20	
Precision (%)	55.66

**Fig. 3:**
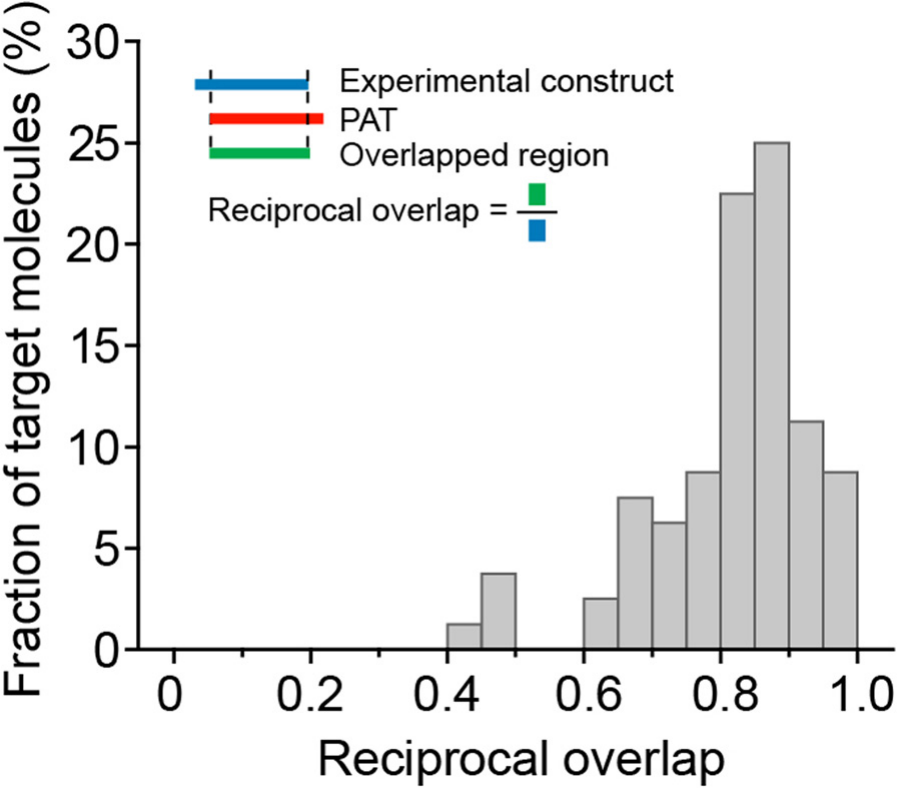
Distribution of the reciprocal overlap between PAT prediction and known experimental constructs that produce synthetic antibodies

Next, we compared predicted targets from PAT with known experimental constructs that are used for synthetic antibody generation. From an in-house pipeline for the generation of synthetic antibodies, we received the sequences of 75 experimentally characterized constructs that are part of protein structures and against which antibodies were successfully produced using phage display (Additional files 1 and 4). We found that PAT can correctly identify these target molecules. Of 75 constructs, PAT correctly predicted 66 (88 %) with a reciprocal overlap greater than 70 % (See Fig. 3 and Additional file 4).

For a comparative performance evaluation of PAT predictions, we also applied DisMeta [7] and DomPred [28] to these 75 experimentally characterized constructs (Additional file 4). We found that PAT outperforms the other two methods. Only 6 constructs (8 %) and 41 constructs (54.67 %) have a reciprocal overlap (>70 %) with DisMeta and DomPred, respectively. Also, the overall reciprocal overlap of PAT (84.43 %, standard deviation ± 9.86) is about 1.5 times higher than overlaps of DisMeta (43.72 %, standard deviation ± 16.25) and DomPred (71.30 %, standard deviation ± 27.36).

### Description of PAT

**Fig. 4:**
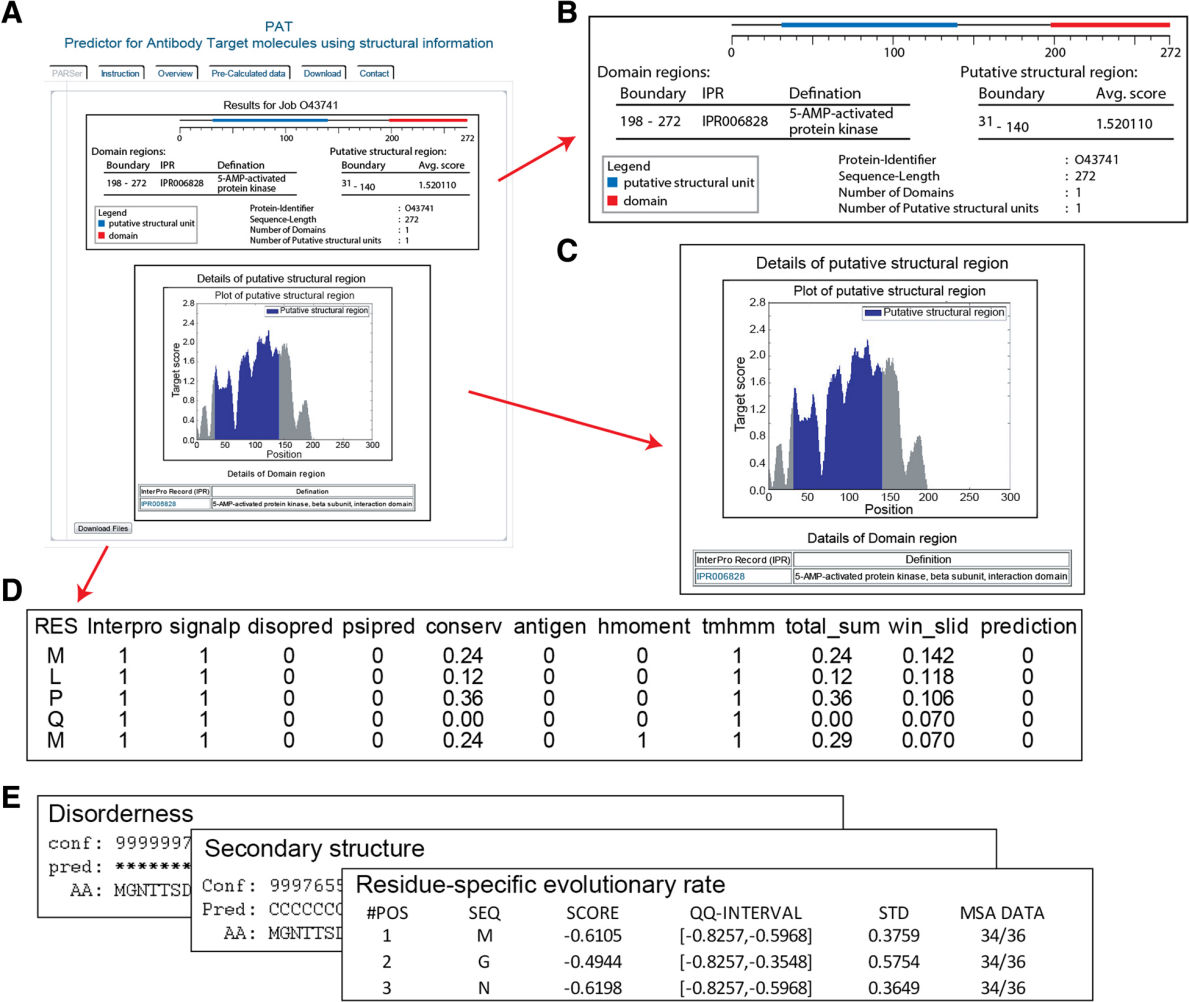
PAT webserver outputs. **a** Output page of PAT. **b** Schematic view and boundary information of structured units. Structured units are colored as red (known domains) and blue (putative structural units). **c** Plot of target score. Putative structural unit is colored as blue. Residues that are not involved in known protein domains are considered to calculate target score. Known protein domain regions are scored as 0. **d** Summarized information of structured units. **e** Intermediate results that are created in the PAT pipeline

PAT provides a list of structural domains with reliable boundaries and related sequence and structural information (Fig. 4a). A vector image visualizes the predicted target molecules (Fig. 4b). The table provides the boundaries of the protein domains including InterPro domain definitions. The average target scores of putative structural units are shown with a score vs residue plot (Fig. 4c). Users can download the summarized information on the results of each analysis together with the predicted target molecules (Fig. 4d) and all intermediate results that are created in PAT pipelines such as disorderness, secondary structure, residue-specific evolutionary rate (Fig. 4e). Furthermore, PAT provides pre-processed antibody target regions for the whole human proteome (20,210 human proteins from UniProt; [29]) to allow for swift lookup of results for frequently accessed proteins. PAT is available as a web server and a downloadable program (http://www.kimlab.org/software/pat).

## Conclusions

The availability of high quality protein structural domains is a necessary prerequisite for protein engineering, protein structure determination and successful antibody generation. PAT is an effective tool to find potential structural domains by adapting a novel integrative scoring scheme and has been shown to do so efficiently. We believe that PAT has great practical value to researches focusing on large-scale structured target production and will ultimately improve the success rate for synthetic antibody generation and follow up studies.

## Availability and requirements

**Project name:** PAT.

**Project home page:**http://www.kimlab.org/software/pat.

**Operating system(s):** Linux for the distributed source code and operating system independent for the web servers.

**Programming language:** Python 2.6 and C++.

**License:** Non-commercial use only.

**Any restrictions to use by non-academics:** Contact authors for permission.

## Additional files

Additional file 1: Methods and additional figures 1–3. (PDF 362 kb) Additional file 2: A list of putative structural units. (XLS 48 kb) Additional file 3: A list of domains that are tested for expression and purification. (XLS 41 kb) Additional file 4: Experimental constructs that successfully produce antibodies. (XLS 37 kb)
