# Supplementary material for: PAT: predictor for structured units and its application for the optimization of target molecules for the generation of synthetic antibodies
Source: BMC Bioinformatics. 2016 Apr 1;17:150. doi: 10.1186/s12859-016-1001-1 (PMC4818438; doi:10.1186/s12859-016-1001-1)
Supplement: Additional file 1: — Methods and additional figures 1–3. (PDF 362 kb) [file 12859_2016_1001_MOESM1_ESM.pdf]

Methods

Identifying protein domains and their boundaries

To identify protein domains with optimized boundaries, PAT defined two types of domains: sequence-based domains and structure-based domains. Sequence-based domains are derived from sequence profiles based on alignments of known domain sequences and structure-based domains are defined from the structural relationship of known domain structures [1]. It has been shown that different methods to delineate the sequence-based domains and structure-based domains complement each other and thus help to capture reliable protein domains with structural boundaries [2]. To identify sequence-based domain, PAT combines the domain information of Pfam [3], SMART [4], and PROSITE [5]. Sequence-based domains are defined if more than 2 databases capture the same region as a domain. Structure-based domains are derived from the domain information of Gene3D [6]. Gene3D is a recently updated database of protein domains and provides comprehensive structural annotation using HMM models based on the CATH domain families [7]. We used the most recently updated version of databases for study.

Identifying putative structural units

To compile structure-related information of putative structural units, PAT uses PSIPRED for secondary structure identification [8], InterProScan to obtain known domain information [9], TMHMM [10] and SignalP 4.0 [11] to examine the presence of trans-membrane and signal peptide and ConSeq to calculate residue-specific evolutionary rate based on the notion that structurally important residues show slow evolutionary rate [12]. DISOPRED2 is used to define ordered and disordered residues in proteins. DISOPRED2 generates position-specific score

matrices by analyzing sequence profiles of homologous sequences and estimate disorder probability of each residue. Then, DISOPRED2 determines structural-state of each residue based on disorder probability (order; disorder probability is smaller than false positive rate threshold and disorder; disorder probability is bigger than false positive rate threshold) [13]. We used result of two-state prediction. To identify antibody-targetable structural regions, PAT integrates antigenicity and hydrophobicity based on the notion that residues such as Cys, Leu and Val are more likely to be a part of antibody recognition sites [14] and hydrophobic residues can affect interactions between target molecules and antibodies [15]. Antigenic and Hmoment from EMBOSS package are used to measure antigenicity and hydrophobicity of residues [16] Antigenic analyses the physicochemical properties of amino acids and their frequencies of occurrence in experimentally known antibody recognition regions and finds antigenic sites in proteins. All tools which are integrated in PAT pipeline are applied with default options. Homologous sequences are selected from Swiss-Prot/TrEMBL to calculate residue-specific evolutionary rate (ConSeq) and to predict disorder (DISOPRED) and secondary structure (PSIPRED). Sequences whose length is 0.7~1.4 times the query sequence length and < 90% similarity to other sequences are considered. It has been shown that PSIPRED and DISOPRED2 show the best performance to predict secondary structures [17] and disordered regions [18], respectively. Therefore, we used their prediction results to characterize putative structural units.

After compiling this information, PAT assigns scores, which represents structure-related properties. Since we focused on the identification of structural regions, we assigned high scores to residues that are involved in secondary structure (0: secondary structure absent, 1: secondary structure present), ordered region (0: disordered residue, 1: ordered residue) and have slow

evolutionary rate (0 to 1 in step size of 0.2. Evolutionary rates below the average are divided into 3 equal intervals. The same 3 intervals are used for the scores above the average. In total, 6 grades were obtained depending on the level of conservation. 0 and 1 indicate low and high conservation, respectively). Furthermore, to consider structural regions that can be recognized by antibodies, we assigned high score to residues that are predicted as antigenic sites (0: non-antigenic site, 1: antigenic site) and tend to be hydrophobic (0: $<$ top 50%, 0.5: top 50% $\leq$ hydrophobic score $\leq$ top 75%, 1: $\geq$ top 75% of hydrophobic scores). Trans-membrane regions, signal peptides, and known domains are excluded for further analysis to find only putative structural unit. Next, we measured the relevance of each structural feature and optimized a scoring scheme. A grid-search method was applied to find the best weight of each structural feature. The weights of each feature were selected ranging from 0.1 to 1.0 with a step size of 0.1 and all possible combinations of weights for features were tested on performance on the training set. As a training set, we used 164 mammalian proteins whose known structures are not listed as domains (putative structural units; Table S1). We collected all solved mammalian protein structures (that would be the part of entire proteins) from PDB database [19] and selected structures that do not have Pfam domain annotations in a given structural region. The training set includes 16,430 residues that are involved in known protein structures (positive set) and 63,122 residues that do not have any structural information such as known domains and structures in 164 proteins (negative set). As a test set, we used 20 protein structures that are not used for the training of PAT pipeline and do not have Pfam domain annotations. The 20 proteins include 3,169 positions that are involved in putative structural regions (positive set) and 5,489 positions that do not have domain annotations and known structures (negative set).

Comparing PAT prediction with experimentally characterized constructs for antibody generation

From an in-house pipeline for the generation of synthetic antibodies, we selected 75 targets that are localized to extracellular regions and against which antibodies were successfully produced using phage display (Additional file 4). The boundaries of these experimental constructs are determined by manual inspection and several experimental trials/errors. We assumed that manually characterized boundaries would be optimized boundaries to get expressed and purified constructs. Among the 75 constructs, 26 contain predicted putative structural units. We compared boundaries of experimental constructs with boundaries of PAT prediction. For the further performance evaluation of PAT prediction, we also applied DisMeta (http://www.wenmr.eu/wenmr/dismeta-disorder-prediction-metaserver) [20] and DomPred (http://bioinf.cs.ucl.ac.uk/psipred/?dompred=1) [8] to 75 experimental constructs.

References

1. Kong L, Ranganathan S: **Delineation of modular proteins: domain boundary prediction from sequence information**. *Briefings in bioinformatics* 2004, **5**(2):179-192.
2. Zhang Y, Chandonia JM, Ding C, Holbrook SR: **Comparative mapping of sequence-based and structure-based protein domains**. *BMC bioinformatics* 2005, **6**:77.
3. Punta M, Coggill PC, Eberhardt RY, Mistry J, Tate J, Boursnell C, Pang N, Forslund K, Ceric G, Clements J *et al*: **The Pfam protein families database**. *Nucleic acids research* 2012, **40**(Database issue):D290-301.
4. Letunic I, Doerks T, Bork P: **SMART 7: recent updates to the protein domain annotation resource**. *Nucleic acids research* 2012, **40**(Database issue):D302-305.
5. Sigrist CJ, de Castro E, Cerutti L, Cuche BA, Hulo N, Bridge A, Bougueleret L, Xenarios I: **New and continuing developments at PROSITE**. *Nucleic acids research* 2013, **41**(D1):D344-347.
6. Lees J, Yeats C, Perkins J, Sillitoe I, Rentzsch R, Dessailly BH, Orengo C: **Gene3D: a domain-based resource for comparative genomics, functional annotation and protein network analysis**. *Nucleic acids research* 2012, **40**(Database issue):D465-471.
7. Sillitoe I, Cuff AL, Dessailly BH, Dawson NL, Furnham N, Lee D, Lees JG, Lewis TE, Studer RA, Rentzsch R *et al*: **New functional families (FunFams) in CATH to improve the mapping of conserved functional sites to 3D structures**. *Nucleic acids research* 2013, **41**(Database issue):D490-498.
8. Buchan DW, Ward SM, Lobley AE, Nugent TC, Bryson K, Jones DT: **Protein annotation and modelling servers at University College London**. *Nucleic acids research* 2010, **38**(Web Server issue):W563-568.
9. Zdobnov EM, Apweiler R: **InterProScan--an integration platform for the signature-recognition methods in InterPro**. *Bioinformatics* 2001, **17**(9):847-848.

10. Moller S, Croning MD, Apweiler R: **Evaluation of methods for the prediction of membrane spanning regions**. *Bioinformatics* 2001, **17**(7):646-653.
11. Petersen TN, Brunak S, von Heijne G, Nielsen H: **SignalP 4.0: discriminating signal peptides from transmembrane regions**. *Nature methods* 2011, **8**(10):785-786.
12. Berezin C, Glaser F, Rosenberg J, Paz I, Pupko T, Fariselli P, Casadio R, Ben-Tal N: **ConSeq: the identification of functionally and structurally important residues in protein sequences**. *Bioinformatics* 2004, **20**(8):1322-1324.
13. Ward JJ, Sodhi JS, McGuffin LJ, Buxton BF, Jones DT: **Prediction and functional analysis of native disorder in proteins from the three kingdoms of life**. *Journal of molecular biology* 2004, **337**(3):635-645.
14. Kolaskar AS, Tongaonkar PC: **A semi-empirical method for prediction of antigenic determinants on protein antigens**. *FEBS letters* 1990, **276**(1-2):172-174.
15. Sundberg EJ, Urrutia M, Braden BC, Isern J, Tsuchiya D, Fields BA, Malchiodi EL, Tormo J, Schwarz FP, Mariuzza RA: **Estimation of the hydrophobic effect in an antigen-antibody protein-protein interface**. *Biochemistry* 2000, **39**(50):15375-15387.
16. Rice P, Longden I, Bleasby A: **EMBOSS: the European Molecular Biology Open Software Suite**. *Trends in genetics : TIG* 2000, **16**(6):276-277.
17. TJ Koswatta PS, VA Sumanasinghe: **A Simple Comparison between Specific Protein Secondary Structure Prediction Tools**. *Tropical Agricultural Research* 2011, **23**(1):91-98.
18. Sirota FL, Ooi HS, Gattermayer T, Schneider G, Eisenhaber F, Maurer-Stroh S: **Parameterization of disorder predictors for large-scale applications requiring high specificity by using an extended benchmark dataset**. *BMC genomics* 2010, **11 Suppl 1**:S15.
19. Sussman JL, Lin D, Jiang J, Manning NO, Prilusky J, Ritter O, Abola EE: **Protein Data Bank (PDB): database of three-dimensional structural information of biological macromolecules**. *Acta crystallographica Section D, Biological crystallography* 1998, **54**(Pt 6 Pt 1):1078-1084.
20. Huang YJ, Acton TB, Montelione GT: **DisMeta: A Meta Server for Construct Design and Optimization**. *Methods in molecular biology* 2014, **1091**:3-16.

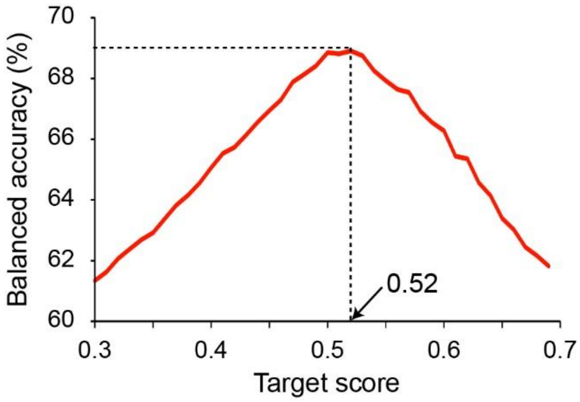

Additional figure 1. Optimization of target score cutoff. Balanced accuracy are shown at the given target score. Balanced accuracy is the arithmetic mean of sensitivity and specificity and avoids inflated performance estimates on imbalanced datasets. The dashed line indicates the cutoff of target score.

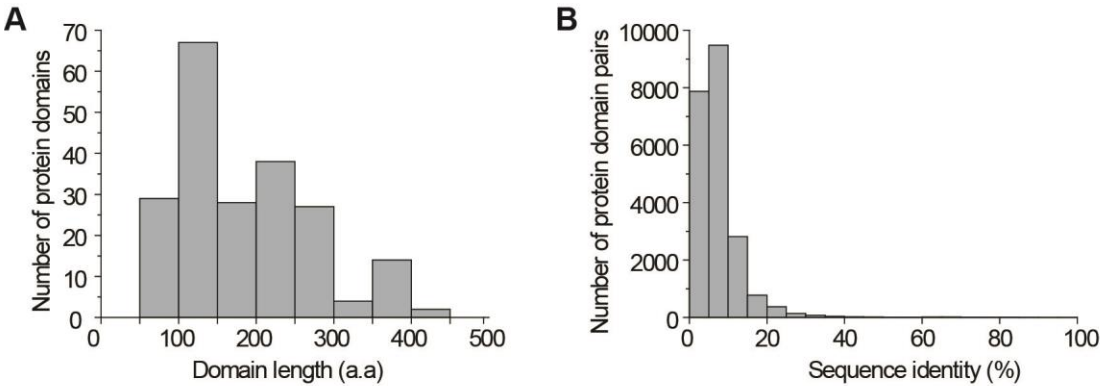

Additional figure 2. The sequence properties of 210 predicted domain regions. Distribution of (A) characterized domain lengths and (B) sequence identities between domain pairs are shown.

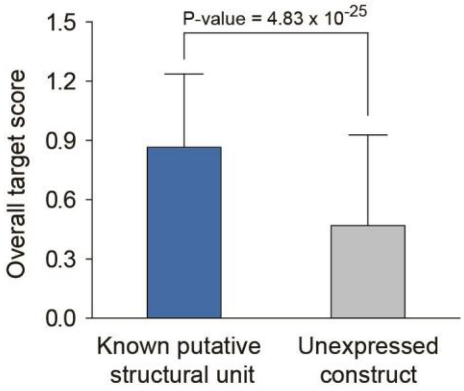

Additional figure 3. Overall target scores of structural regions. Average target scores of known putative structural units (blue bar) and those of unexpressed constructs in TargetTrack (gray bar) are compared.
